# Identifying the optimal ratio from protein foods for protein and nutrient quality in plant-based meals using a non-linear optimization approach

**DOI:** 10.3389/fnut.2025.1624633

**Published:** 2025-10-01

**Authors:** Maryann R. Rolands, Fabio Mainardi, Murielle Bochud, Kim-Anne Lê

**Affiliations:** ^1^University of Lausanne, Lausanne, Switzerland; ^2^Nestlé Institute of Health Sciences, Nestlé Research, Lausanne, Switzerland; ^3^Department of Epidemiology and Health Systems, Unisanté, University Center for Primary Care and Public Health, Lausanne, Switzerland

**Keywords:** plant-based diet, vegetarian, vegan, semi-vegetarian, protein source, plant protein, animal protein, protein quality

## Abstract

**Background:**

Plant-based diets with reduced animal protein intake are increasingly recommended for health and sustainability reasons that have potential implications for nutrient intake, including protein quality.

**Objective:**

To develop a non-linear optimization model to determine the optimal ratio needed from plant and animal protein foods to obtain a high protein and nutrient quality in primarily plant-based meals.

**Methods:**

Sixty-two protein foods were grouped by their limiting amino acid: “lysine-limiting” foods were mainly “grains, nuts and seeds,” “sulfur amino acids” mainly consisted of “beans, peas and lentils” and “non-limiting” included “soy-foods” for vegan and vegetarian meals and/or “animal protein foods” for pesco/semi-vegetarian meals.” A non-linear optimization approach was used to maximize protein quality using the Protein Digestibility Corrected Amino Acid Score (PDCAAS 1) while considering essential nutrients such as energy, protein, dietary fiber, iron, calcium, and zinc in three plant-based meal models. The three models considered all contained protein foods from the first two groups, “grains, nuts and seeds” and “beans, peas, lentils, and others” and some variations of the third group being either “soy-foods only” (vegan), “soy-foods, dairy and egg” (vegetarian), or “soy-foods and/or animal-based foods” (pesco/semi-vegetarian).

**Results:**

To achieve optimal protein quality, calcium, iron, and zinc levels in a vegan and vegetarian meal, the optimal protein ratio based on total protein intake was at least 10% grains, nuts, and seeds; 10–60% beans, peas, and lentils; and 30–50% soy-based foods only and/or dairy and eggs. The optimal pesco/semi-vegetarian meal had at least 10% grains, nuts, and seeds, 50–60% beans, peas, and lentils, and 30–40% from soy-foods and/or animal-based foods. The vegan meal had more variety than models including animal protein foods.

**Conclusion:**

The optimal ratios of protein foods determined could be used to define easy-to-follow guidance for selecting protein foods that deliver high protein quality while also contributing to nutrient quality in primarily plant-based meals.

## 1 Introduction

Plant-based meals and diets with reduced intake from animal-based foods, especially in high-income countries, are increasingly recommended for health and sustainability reasons and may have potential implications for overall diet quality and nutrient intakes, including protein quality (1, 2). Foods contain proteins that consist of two main types of amino acids: (i) dispensable amino acids, which the human body can synthesize on its own, and (ii) indispensable amino acids (IAA), which the human body cannot synthesize and must obtain from the diet (3). Apart from consuming sufficient protein, the quality of protein consumed in the human diet is also important as it considers how well protein is digested and absorbed for use by the body. Protein quality consists of amino acid composition, protein content, individual amino acid digestibility, and the requirement of IAA in the target population (3). The protein digestibility amino acid score (PDCAAS) is the most widely used protein quality metric and is based on the first limiting amino acid relative to a reference pattern (3, 4). High-quality protein foods such as meat, poultry, fish, eggs, certain soy-based foods, and dairy foods (milk, cheese, and yogurt) provide all the 9 IAA and are also carriers of several key nutrients such as iron, zinc, calcium, vitamin B12, and phosphorus (3, 4). Although plant protein foods such as legumes, grains, nuts, seeds, and vegetables can be deficient in one or more IAA, these foods contribute highly to dietary fiber, vitamin E, and magnesium intakes (5). Therefore, for individuals mainly relying on plant protein foods, high-quality protein, and other nutrient intakes can only be achieved by specific combinations of plant and animal protein foods, allowing them to compensate for the various limiting amino acids and key nutrients (6). For example, a study that simulated flexitarian dietary patterns, which involved reducing animal protein intake from 75 to 25%, revealed higher Healthy Eating Index-2015 scores with reduced animal protein intake as compared to an omnivorous diet that consisted of over 75% of animal protein (7).

Existing dietary recommendations from several Western and Asian populations emphasize consuming more plant proteins (50–75%) without specifying proportions from the different types of plant-protein foods (8–11). Our previous work has shown that individuals following a semi-vegetarian or flexitarian diet reducing animal protein foods tended to consume mainly plant protein foods such as grains, nuts, and seeds and least from beans, peas, lentils, and soy-based foods (12). Diet modeling analyses of national dietary surveys in the United States and Canada found that replacing amino acids from grain protein with those from legumes, lentils, or non-grain plant proteins increased overall protein quality as well as levels of fiber, folate, iron, and zinc in the diet (13, 14). Mainly relying on cereal-based foods as a plant protein food instead of incorporating a variety from other high-quality plant-protein foods such as beans, peas, lentils, and soy-based foods could affect protein quality and other key nutrient intakes (12).

Currently, most dietary guidelines are clear on including a variety of protein foods in meals and diets for optimal protein and overall diet quality (8–11). However, it remains uncertain which types of plant protein foods should be consumed and in which proportions. Consequently, it becomes important to define easy-to-follow dietary guidance for the general population aiming to reduce animal-based foods by selecting foods delivering high protein quality while contributing to nutrient quality to help reach a balanced primarily plant-based meal. The main aim of this study is to develop a non-linear optimization method to determine optimal ratios of plant and animal protein foods to obtain a high protein and nutrient quality in primarily plant-based meals (vegan, vegetarian, and pesco, or semi-vegetarian).

## 2 Methods

### 2.1 Data origin

All cooked and ready-to-eat plant protein foods (*N* = 51) and animal protein foods (*N* = 11) had their IAA, macronutrients, and micronutrients information extracted from the US Department of Agriculture (USDA) national nutrient database for standard reference release 28, considering that the preparation method could influence nutrient availability and also to reflect the typical consumption of foods; for example, almonds are usually consumed raw, while rice is cooked (15) (Table 1; Supplementary Table 1). The protein digestibility values, and processing method of each food item were obtained from various sources of the literature as indicated in Table 1 and Supplementary Table 1. There was an exception with three ingredients (soy milk, seitan, and carrots), where the total protein content, IAA information, and protein digestibility values were obtained from the literature alone (16, 17). The selection process for the 62 foods has been described in Supplementary Figure 1.

**Table 1:** Information extracted on protein foods (*N* = 62) from the USDA national database standard reference release 28 (15).

Macronutrients	Micronutrients	Indispensable amino acids
Energy (Kcal/100 g) Total carbohydrate (g/100 g) Dietary fiber (g/100 g) Total protein (g/100 g) Total fat (g/100 g) Saturated fat (g/100 g)	Iron (mg/100 g) Calcium (mg/100 g) Zinc (mg/100 g)	Isoleucine (g/100 g) Leucine (g/100 g) Lysine (g/100 g) Methionine (g/100 g) Cysteine (g/100 g) Phenylalanine (g/100 g) Tyrosine (g/100 g) Threonine (g/100 g) Tryptophan (g/100 g) Valine (g/100 g) Histidine (g/100 g)

As shown in Table 2, the limiting amino acid was identified as the lowest amount of IAA present in the protein food. The amino acid score assesses how effectively the absorbed dietary nitrogen can fulfill the IAA requirements at a safe level of protein consumption and was calculated by comparing the limiting amino acid content (mg per g) in each protein food with the amino acid requirement pattern (mg per g) (18). The protein digestibility of protein foods was measured by how efficiently a protein food is digested and absorbed in the body while also considering protein that would be lost through the feces. The protein digestibility values of the included plant and animal protein foods in this study were extracted from the literature (Table 2), prioritizing human ileal digestibility where available, followed by digestibility from studies in pigs and rats, and finally values from *in vitro* models if needed (4). The PDCAAS value for each protein food was calculated by multiplying the amino acid score with the protein digestibility value.

**Table 2:** Plant protein foods with amino acid score, protein digestibility %, food processing method used, species protein digestibility tested *in vivo* or *in vitro* method and calculated PDCAAS.

Protein food	Limiting amino acid	Amino acid score	Protein digestibility (%)	PDCAAS	Processing method	Species/*in vitro*	*In vivo* / *in vitro* model	Reference
Barley pearl cooked	Lysine	0.77	78	0.6	Cooked	Human	Ileal	(43)
Coconut milk, canned	Lysine	0.84	54	0.45	Extracted	Human	Ileal	(44)
Oat bran, raw	Lysine	0.89	74	0.66	Raw	Human	Ileal	(43)
Pasta, whole-wheat, cooked	Lysine	0.46	92	0.42	Cooked	Human	Ileal	(43)
Bread, whole-wheat	Lysine	0.61	91	0.55	Processed as bread	Human	Ileal	(43)
Kidney beans, cooked, boiled	SAA	1.18	74	0.87	Soaked for 12 h. at 25 °C and 70 min cooked	Human	Ileal	(43)
Lupins, cooked, boiled	SAA	0.83	90	0.75	Processed as flour	Human	Ileal	(45)
Mung beans, cooked, boiled	SAA	0.93	63.2	0.59	Soaked for 12 h. and cooked	Human	Ileal	(46)
Yellow peas, split, cooked, boiled	SAA	1.06	71.6	0.76	Soaked for 12 h. and pressure cooked	Human	Ileal	(46)
Potatoes, French fried	SAA	0.81	51	0.41	Potato fries (fat 12–18%)	Human	Ileal	(43)
Chickpeas, cooked, boiled	Valine	1.09	74.6	0.81	Soaked for 12 h. and cooked in a curry	Human	Ileal	(46)
Egg, whole, boiled	Non-limiting	1.41	89.4	1	Boiled for 20 min	Human	Ileal	(47)
Chicken, breast, skinless, boneless, meat only, cooked	Non-limiting	1.33	92	1	The meat was cooked for 40 min	Human	Ileal	(47)
Cheese, gouda	Non-limiting	1.27	88.5	1	Cheese	Human	Ileal	(43)
Fish, whitefish, cooked	Non-limiting	1.34	91	1	Fish meal	Human	Ileal	(43)
Bread, pita, white	Lysine	0.5	94.7	0.47	Baked into bread	Pig	Ileal	(48)
Millet, cooked	Lysine	0.4	80.2	0.32	Soaked for 30 min at 25 °C then cooked	Pig	Ileal	(48)
Oat bran, cooked	Lysine	0.89	87.4	0.78	Soaked for 30 min at 25 °C then cooked	Pig	Ileal	(48)
Rice, brown, long-grain, cooked	Lysine	0.8	84.4	0.67	Soaked for 30 min at 25 °C then cooked	Pig	Ileal	(48)
Adzuki beans, cooked, boiled	SAA	0.91	75.9	0.69	Soaked for 12 h. at 25 °C and 70 min cooked	Pig	Ileal	(49)
Broad beans (fava beans), cooked, boiled	SAA	0.97	77.9	0.76	Soaked for 12 h. at 25 °C and 70 min cooked	Pig	Ileal	(49)
Buckwheat groats roasted, cooked	Non-limiting	1.14	88.2	1	Soaked for 30 min at 25 °C then cooked	Pig	Ileal	(48)
Tofu	Non-limiting	1.63	95	1	Raw	Pig	Ileal	(16)
Soya milk	Non-limiting	1.27	92	1	UHT milk	Pig	Ileal	(16)
Milk, dry, nonfat, regular	Non-limiting	1.42	92	1	Powdered	Pig	Ileal	(38)
Beef, loin, top loin steak, boneless	Non-limiting	1.22	98	1	Topside, steak	Pig	Ileal	(50)
Beef, cured, dried	Non-limiting	1.02	98	1	Beef jerky	Pig	Ileal	(50)
Pork loin, cooked-roasted, lean	Non-limiting	1.28	99	1	Pork loin cooked to medium and well done	Pig	Ileal	(51)
Pork, leg (ham), cooked, roasted	Non-limiting	1.28	98	1	Pork conventional ham	Pig	Ileal	(51)
Pork, cured, bacon, cooked, pan-fried	Non-limiting	1.28	98	1	Pork smoked bacon	Pig	Ileal	(51)
Salami, Italian, pork	Non-limiting	1.19	96	1	Salami preparation	Pig	Ileal	(52)
Peanuts, all types, raw	Lysine	0.74	90.9	0.67	Roasted	Rat	Ileal	(53)
Rice, white, long-grain, cooked	Lysine	0.75	72.8	0.55	Cooked in rice cooker	Rat	Ileal	(53)
Cereals ready-to-eat, Corn Flakes	Lysine	0.24	67	0.16	Corn flakes	Rat	Ileal	(43, 53)
Potatoes, boiled without skin	Leucine	0.98	95	0.93	Cooked	Rat	Fecal	(54)
Carrots, cooked, boiled, drained	Leucine	0.98	80	0.78	Cooked	Rat	Fecal	(54)
Almond butter	Lysine	0.57	88.9	0.51	Raw	Rat	Fecal	(55)
Almonds raw	Lysine	0.52	88.9	0.46	Raw	Rat	Fecal	(55)
Nuts, cashew butter, plain	Lysine	1.01	87.7	0.89	Raw	Rat	Fecal	(56)
Nuts, cashew nuts, raw	Lysine	0.97	87.7	0.85	Raw	Rat	Fecal	(56)
Oats rolled	Lysine	0.87	88	0.76	Raw	Rat	Fecal	(43)
Nuts, walnuts, black, dried	Lysine	0.6	86.2	0.52	Raw	Rat	Fecal	(57)
Walnuts, English	Lysine	0.56	86.2	0.48	Raw	Rat	Fecal	(57)
Fried seitan coated with wheat flour	Lysine	0.42	77.3	0.32	Fried using sunflower oil at 170 °C for 20 s	Rat	Fecal	(58)
Black beans, cooked, boiled	SAA	1.19	70	0.83	Soaked for 16 h. and boiled 18.5 min	Rat	Fecal	(59)
Split peas, cooked, boiled	SAA	1.16	85.2	0.98	No soaking and boiled for 34 min	Rat	Fecal	(59)
Navy beans, cooked, boiled	SAA	0.97	80	0.78	Soaked for 16 h. and boiled 18.6 min	Rat	Fecal	(59)
Pinto beans, cooked, boiled	SAA	0.98	76.2	0.75	Soaked for 16 h. and boiled for 19.2 min	Rat	Fecal	(59)
Lentils, cooked, boiled	SAA	1.02	90.6	0.92	No soaking and boiled for 12 min	Rat	Fecal	(59)
Apples, raw with skin	SAA	0.41	52	0.21	Raw	Rat	Fecal	(54)
Kale, cooked, boiled, drained	SAA	1.1	77	0.85	Cooked	Rat	Fecal	(54)
Soybeans, cooked, boiled	Non-limiting	1.18	94	1	Soaked for 24 h. and boiled for 180 min	Rat	Fecal	(60)
Chia seeds, dried	Lysine	1.04	29	0.3	Raw	*In Vitro*	Pepsin only	(61)
Pumpkin and squash seed kernels	Lysine	0.77	90	0.69	Raw	*In Vitro*	Multienzyme	(62)
Sesame seed kernels, toasted	Lysine	0.59	75	0.44	Raw	*In Vitro*	Multienzyme	(62)
Sunflower seed kernels, dried	Lysine	0.78	90	0.7	Raw	*In Vitro*	Multienzyme	(18)
Watermelon seed kernels, dried	Lysine	0.58	63	0.37	Raw	*In Vitro*	Multienzyme	(63)
Tortillas, ready-to-bake or -fry, corn	Lysine	0.59	84	0.49	Ready to bake/fry	*In Vitro*	Multienzyme	(64)
Mushrooms, portabella, raw	SAA	0.95	81.6	0.77	Raw	*In Vitro*	INFOGEST	(65)
Cowpeas, catjang, cooked, boiled	Non-limiting	1.11	98	1	Boiled for 35 min	*In Vitro*	Multienzyme	(66)
Tofu, fried	Non-limiting	1.2	89.6	1	Fried (170 °C, 20 s)	*In Vitro*	Multienzyme	(58)
Yeast (*Saccharomyces cerevisiae*)	Non-limiting	1.21	82.9	1	Processed as a concentrate	*In Vitro*	INFOGEST	(67)

### 2.2 Protein foods categorization approach

**Figure 1:**
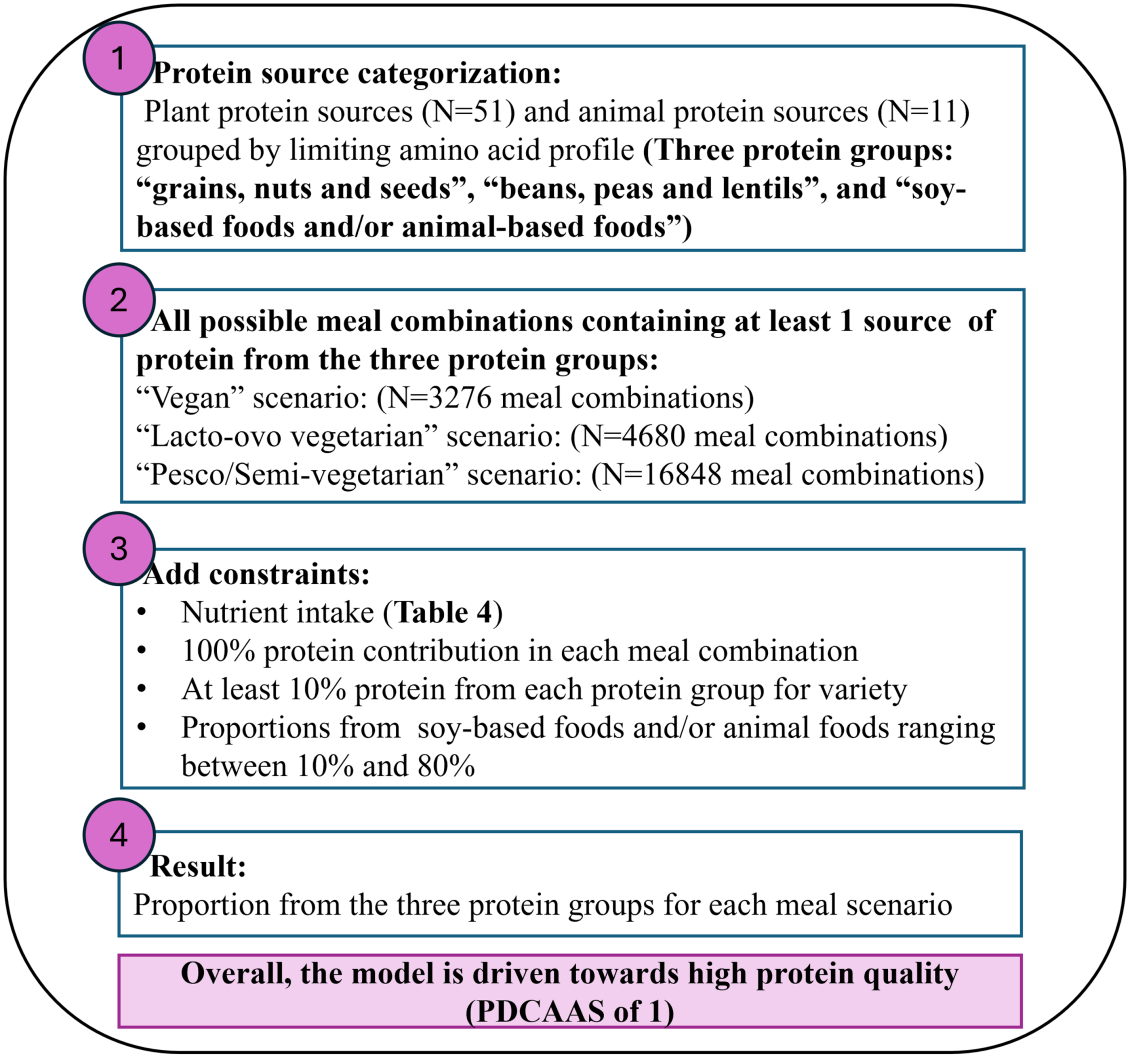
Overview of the non-linear model process to obtain the optimal protein ratios from the three protein food groups based on limiting amino acid profile.

In step 1 of Figure 1, the fifty-one plant protein foods and eleven animal protein foods were grouped by protein food groups according to their limiting amino acid profile. Based on the protein foods and their corresponding limiting amino acid as shown in Table 2. The protein food groups were defined as “lysine-limiting,” “sulfur amino acids (SAA)-limiting” and “non-limiting” which mainly consisted of “grains, nuts and seeds,” “beans, peas, lentils and others” and “soy-based foods only and/or animal foods and others,” respectively (Table 3). A few protein foods fell into groups that would differ from the corresponding food group. Examples include peanut and coconut (botanically classified as legumes and fruit, respectively), which are categorized as “lysine limiting.” Similarly, apples, carrots, kale, and potatoes (i.e., classified as fruit and vegetables) were grouped under “SAA-limiting,” whereas buckwheat, cowpeas, and yeast (*Saccharomyces cerevisiae*) had “non-limiting” amino acid profiles, thus grouped as a “non-limiting” protein food. Foods such as boiled potatoes and carrots were “leucine-limiting” and chickpeas “valine-limiting”; they grouped under “SAA-limiting” as the methionine and cysteine levels were reported to be the second lowest amino acids present, with carrots reflecting levels below the reference intake for methionine and cysteine in adults.

**Table 3:** Plant protein foods are grouped according to their limiting amino acid profile based on Table 2.

Lysine-limiting (*N* = 26)	SAA-limiting (*N* = 18)	Non-limiting (*N* = 18)
“Grains, nuts and seeds”	“Beans, peas, lentils and others”	“Soy-based foods only and/or animal foods and others”
Barley pearl cooked	Kidney beans, cooked, boiled	Buckwheat groats roasted, cooked
Bread, pita, white	Adzuki beans, cooked, boiled	Cowpeas, catjang, cooked, boiled
Almond butter	Black beans, cooked, boiled	Soybeans, cooked, boiled
Almonds raw	Chickpeas, cooked, boiled	Tofu
Nuts, cashew butter, plain	Broad beans (fava beans), cooked, boiled	Tofu, fried
Nuts, cashew nuts, raw	Split peas, cooked, boiled	Soya milk
Seeds, chia seeds, dried	Lupins, cooked, boiled	Yeast (*Saccharomyces cerevisiae*)
Coconut milk, canned	Mung beans, cooked, boiled	Egg, whole, cooked, fried*
Millet, cooked	Navy beans, cooked, boiled	Milk, dry, nonfat, regular*
Oat	Pinto beans, cooked, boiled	Chicken, breast, meat only, cooked*
Oat bran, raw	Potatoes, boiled without skin	Cheese, gouda*
Oat bran, cooked	Lentils, cooked, boiled	Fish, whitefish, cooked*
Peanuts, raw	Pigeon peas, cooked, boiled	Beef, loin, top loin steak, boneless*
Pumpkin and squash seed kernels, dried	Mushrooms, portabella, raw	Beef, cured, dried*
Rice, brown, long-grain, cooked	Carrots, cooked, boiled, drained	Pork loin, cooked-roasted, lean*
Rice, white, long-grain, regular, cooked	Apples, raw with skin	Pork, leg (ham), cooked, roasted*
Sesame seed kernels, toasted	Kale, cooked, boiled, drained	Pork, cured, bacon, cooked, pan-fried*
Sunflower seed kernels, dried	Potatoes, French fried	Salami, Italian, pork*
Walnuts, black, dried		
Walnuts, English		
Watermelon seed kernels, dried		
Pasta, whole-wheat, cooked		
Fried seitan coated with wheat flour		
Tortillas, ready-to-bake or -fry, corn		
Bread, whole-wheat		
Cereals ready-to-eat, Corn Flakes		

*Animal protein foods include eggs, milk, cheese, chicken, fish, beef, and pork.

### 2.3 Non-linear optimization programming approach

The non-linear optimization approach is a mathematical methodology used to find the best solution from a set of possible solutions where the objective function or the defined constraints are non-linear (19). Although the Digestible Indispensable Amino Acid Score (DIAAS) is a more accurate measurement of protein quality, the data available for a variety of foods remains limited. Thus, in this study. PDCAAS was considered as the most appropriate measurement for protein quality. The key aspects covered in a non-linear optimization model include an objective function, which in this study aims to maximize the PDCAAS to enhance protein quality in a meal. The decision variables in this model represent the proportions of different foods from the respective food groups included in the meal. Additionally, the model includes constraints that define a feasible region within which the optimal solution for protein quality and nutrient content in a meal can be identified. In this study, these constraints relate to the determined nutrient contributions of the meal. As shown in Figure 1, a non-linear optimization model using R package NlcOptim was built to maximize the objective function [PDCAAS = (amino acid score × protein digestibility %)] between all meal combinations of the three protein food groups (Table 3). In step 2 of Figure 1, all meal combinations were determined in three primarily plant-based meal scenarios across the three protein food groups (Table 3). The first meal category was defined as a typical “vegan” meal using foods from (i) “grains, nuts and seeds,” (ii) “beans, peas, lentils and others” and (iii) “soy-based foods and others” creating 3,276 unique meal combinations. The second meal category was defined as a typical “vegetarian” meal using foods from (i) “grains, nuts, and seeds,” (ii) “beans, peas, lentils, and others” and (iii) “soy-based foods and inclusion of dairy and/or eggs” creating 4,680 unique meal combinations. Finally, the last meal category was defined as a typical “pesco/semi-vegetarian” meal using foods from combinations of (i) “grains, nuts, and seeds,” (ii) “beans, peas, lentils, and others” and (iii) “soy-based foods and inclusion of dairy, eggs, fish, poultry and red meats” creating 16,848 unique meal combinations. The detailed calculations to obtain a PDCAAS for each meal combination from the three protein food groups are shown in Supplementary Table 2.

### 2.4 Constraints added to non-linear optimization model

In step 3 of Figure 1, the model was restricted to providing solutions that resulted in a total contribution of 100% protein intake from all protein food groups for each meal combination (Table 3). A constraint of at least 10% was determined to come from the “grains, nuts and seeds” and “beans, peas, lentils, and others” groups to allow for a variety of consumption from all protein food groups (1, 20). Finally, constraints ranging from 10 to 80% were determined on the “soy-based foods and others” or “soy, animal-based foods, and others” protein food group to prevent the model from favoring a solution that consisted entirely of soy or animal protein foods as all foods within these groups had a PDCAAS of 1. The nutrient constraints were selected based on critical nutrients found to be low in plant-based diets (21). Vitamin B12 and vitamin D are essential nutrients for plant-based meals and diets. However, they were not considered due to plant protein foods having no vitamin B12 present, and there was a lack of data available for vitamin D in selected foods.

Dietary intake values were determined using the International Breakfast Initiative nutrient proposal for a breakfast meal in Western Europe and North America to determine an ideal contribution of nutrients from plant protein foods in a plant-based meal (22). Breakfast was used as an example meal for several reasons, including the availability of nutrient guidance for this meal and the daily energy intake recommendations being generally lower for breakfast, allocating 20–25% of total intake thus allowing for adaptation based on individual snacking habits (23). The average-sized breakfast of 317 g was determined by assessing the 20 breakfast recipes listed under the International Breakfast Initiative (24). As shown in Table 4, the range for each nutrient constraint was determined based on nutrient recommendations per 100 g breakfast to an average-sized breakfast of 317 g. As protein intake is a driving factor for optimal protein quality, the total protein intake recommendations were set at a maximum of 30 g per meal to maintain muscle protein synthesis and long-term muscle mass and function (25).

**Table 4:** Nutrient constraints applied to the non-linear model.

Nutrients	Nutrient recommendations based on per 100 g breakfast meal	Nutrient recommendations based on an average breakfast meal (317 g) (22)
Energy (Kcal)	≥ 95	≤ 500
Protein (%)	≥ 3.2	≤30g^a^
Dietary fiber (g)	≥ 1.5	≤ 5 (20% of 25 g)
Iron (mg)	≥ 0.9	≤ 2.8 (20% of 14 mg)
Calcium (mg)	≥ 79	≤ 250 (25% of 1,000 mg)
Zinc (mg)	≥ 0.7	≤ 2.2 (20% of 11 mg)

^a^Total protein intake recommendations were set at a maximum of 30 g per meal in order to maintain muscle protein synthesis, and long-term muscle mass and function (25).

Plant-based foods naturally contain high levels of antinutrients such as phytates which impede the bioavailability of key nutrients such as iron and zinc as well as protein digestibility (26). Therefore, the phytate for each protein food was sourced from the FAO/INFOODS/IZiNCG Global food composition database (27). The phytate-to-iron and phytate-to-zinc molar ratio were calculated for each plant-based meal combination to assess the potential bioavailability of iron and zinc (28).

Higher ratios indicate a greater presence of more phytate to mineral intake. The WHO and FAO guidance on phytate ratios states if there are no iron absorption enhancers like vitamin C consumed in a meal, the phytate-to-iron molar ratio should be aimed to be < 1:1 (low impact on iron absorption) or phytate-to-zinc molar ratio < 5 to 15 (low to moderate impact on zinc absorption) (29). Furthermore, FAO/IZiNCG states a molar ratio >18 would adversely affect zinc absorption (27).

The optimal solution would be defined as high protein quality (PDCAAS 1) and meet the defined target nutrient quality. In step 4 of Figure 1, the non-linear optimization model aimed to maximize the PDCAAS for every combination of a plant-based meal, thereby producing solutions that represented the percentage contribution of total protein intake from each protein group and considered the quality of selected macro and micronutrients. The algorithm did not converge for some combinations of a plant-based meal, thus making these meal combinations “non-applicable.” From all the potential optimal solutions for each meal combination, we selected the most frequent output from the three protein groups in combination with most mixes having a PDCAAS of 1. The distribution of PDCAAS, nutrient intake, and phytate to iron and zinc ratios across all protein ratio solutions comparing vegan, vegetarian, and pesco/semi-vegetarian meals were presented as boxplots. The boxplots display the minimum, interquartile range, median, and maximum for each plant protein food, and any outliers were represented as black dots. The R script used is available here: Mrolands123/GitHub.

## 3 Results

**Figure 2:**
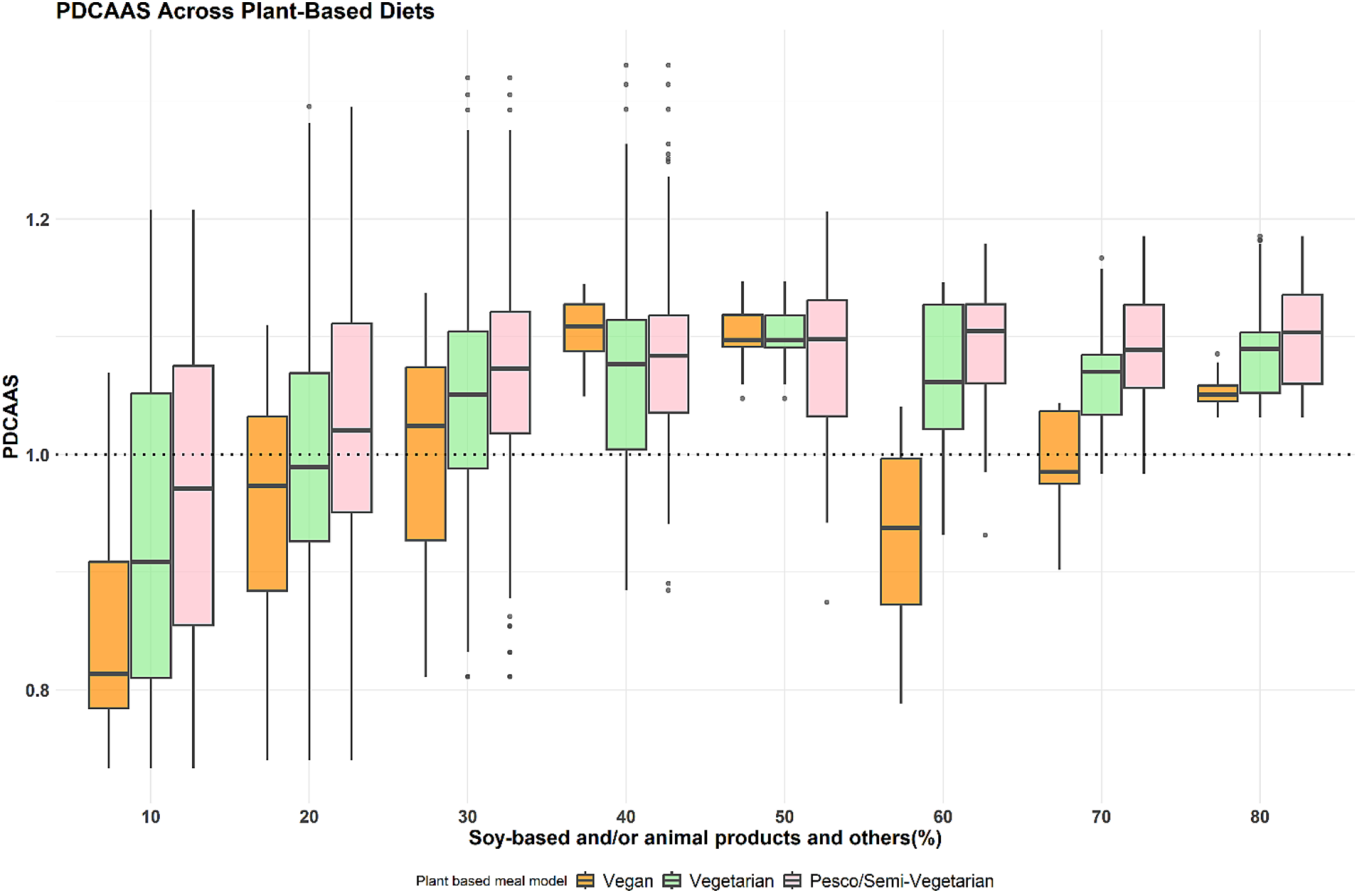
PDCAAS distribution across the three plant-based meal models at different protein food contributions. These boxplots display the minimum, interquartile range, median, and maximum PDCAAS for the percentage contribution of soy-based only and/or animal products to total protein intake across three primarily plant-based meals: vegan, vegetarian, and pesco/semi-vegetarian. The left whisker indicates the minimum contribution, while the upper and lower whiskers represent the lower and upper 25% of intake values, respectively. The box shows the interquartile range, encompassing the middle 50% of intakes (25th to 75th percentile), with the median marked by a line within the box. The right whisker denotes the maximum contribution, and any outliers are displayed as black dots outside the whiskers. The dotted line represents PDCAAS 1 (optimal protein quality). The green box highlights the protein ratios with a median PDCAAS 1 across all three primarily plant-based meals.

As presented in Figure 2, the protein quality (PDCAAS) distribution to achieve optimal protein quality (PDCAAS 1) indicates that most meal combinations must have contributions of at least 30 to 50 and 80% from soy-based foods for vegan meals. The results were similar when including dairy and/or eggs for vegetarian meals, and animal protein foods for pesco/semi-vegetarian meals.

At this optimal ratio for protein quality, the macronutrient distribution indicated that, on average, per 100 g, the energy content ranged from 158 to 195 kcals. The percentage of energy from carbohydrates varied between 26 and 41%, while dietary fiber content ranged from 2.8 to 3.7 g. The proportion of energy derived from protein was between 21 and 24%, and total fat contributed 43 to 57%, with saturated fat accounting for 7 to 8% of energy (Supplementary Figure 2). With 80% of protein foods from soy-based foods only and/or animal protein, the macronutrient distribution across the different plant-based diets showed that, on average, per 100 g, the energy content ranged from 100 to 185 kcals. The percentage of energy from carbohydrates varied between 15.5 and 30.9%, while dietary fiber content ranged from 4.1 to 4.2 g. Protein contributed 27.4 to 32% of energy, and total fat accounted for 40 to 56% of energy. Notably, saturated fat at 80% soy-based foods and/or animal protein contributed 4.9 to 13.7% of energy.

**Figure 3:**
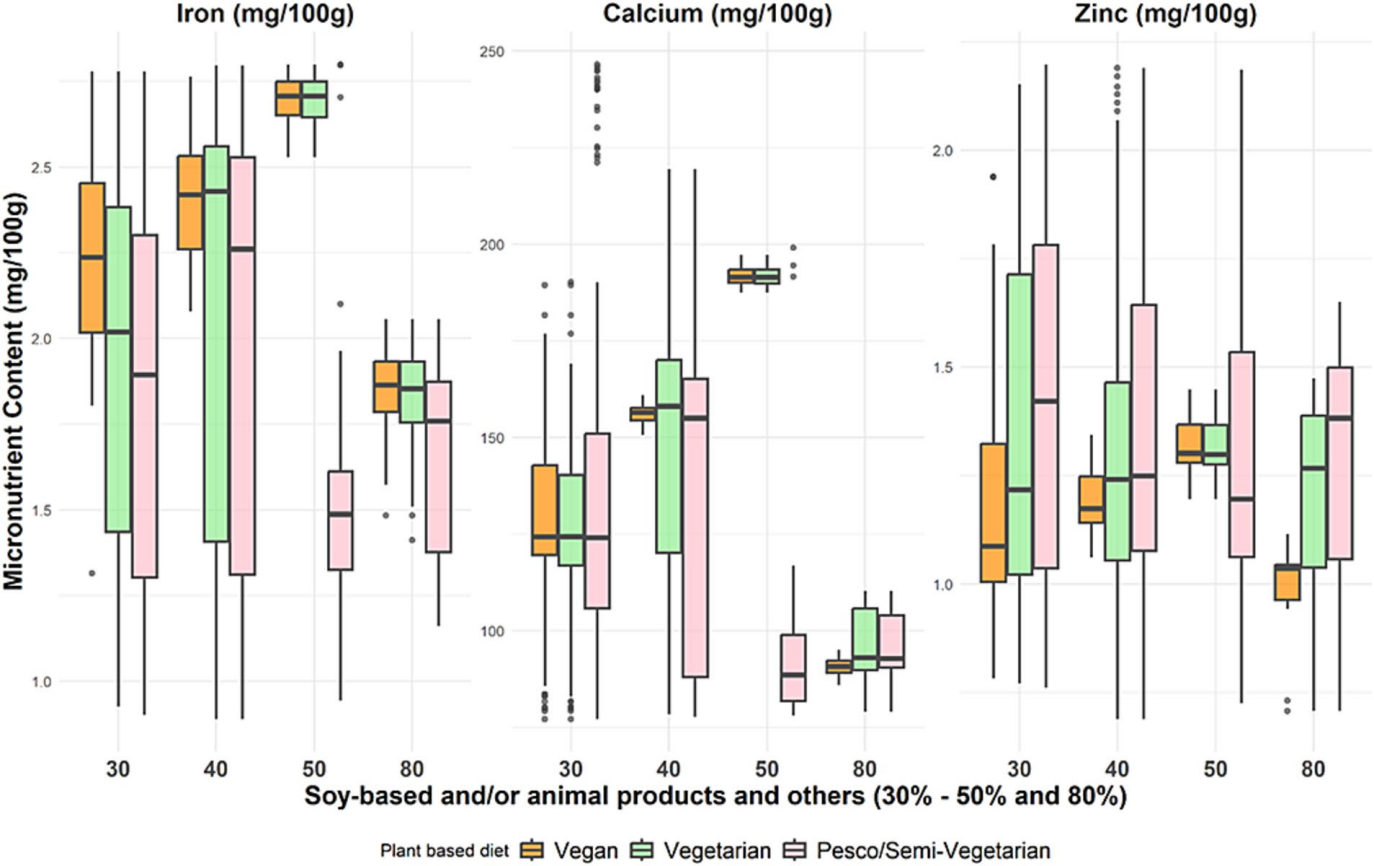
Distribution of selected micronutrient content across the three plant-based meal models at different protein food contributions. These boxplots display the minimum, interquartile range, median, and maximum iron, zinc, and calcium levels for the percentage contribution of soy-based only and/or animal products to total protein intake across three primarily plant-based meals: vegan, vegetarian, and pesco/semi-vegetarian. The left whisker indicates the minimum contribution, while the upper and lower whiskers represent the lower and upper 25% of intake values, respectively. The box shows the interquartile range, encompassing the middle 50% of intakes (25th to 75th percentile), with the median marked by a line within the box. The right whisker denotes the maximum contribution, and any outliers are displayed as black dots outside the whiskers.

At optimal ratios for protein quality (PDCAAS 1), with 30 to 50% protein from soy-based foods only and/or animal protein, the micronutrient distribution in the vegan and vegetarian models showed that, on average, per 100 g, the iron content ranged from 1.85 to 2.71 mg, calcium content ranged from 90.7 to 191.5 mg, and zinc content ranged from 1.03 to 1.3 mg (Figure 3). Whereas at 50% soy and/or animal protein foods contribution, the pesco/semi-vegetarian meal model had the lowest contribution from iron (1.48 mg), calcium (88.5 mg), and zinc (1.19 mg). Therefore, regarding iron and calcium distribution, the optimal soy and/or animal protein contribution in a pesco/semi-vegetarian meal would be 30 to 40%. The distribution of iron, calcium, and zinc intake across the plant-based meals was lowest at 80% contribution from soy and/or animal protein. In addition to iron, zinc and calcium, vitamin B12 was also observed to increase when animal protein foods are included as a solution in either a vegetarian or pesco/semi-vegetarian meal.

**Figure 4:**
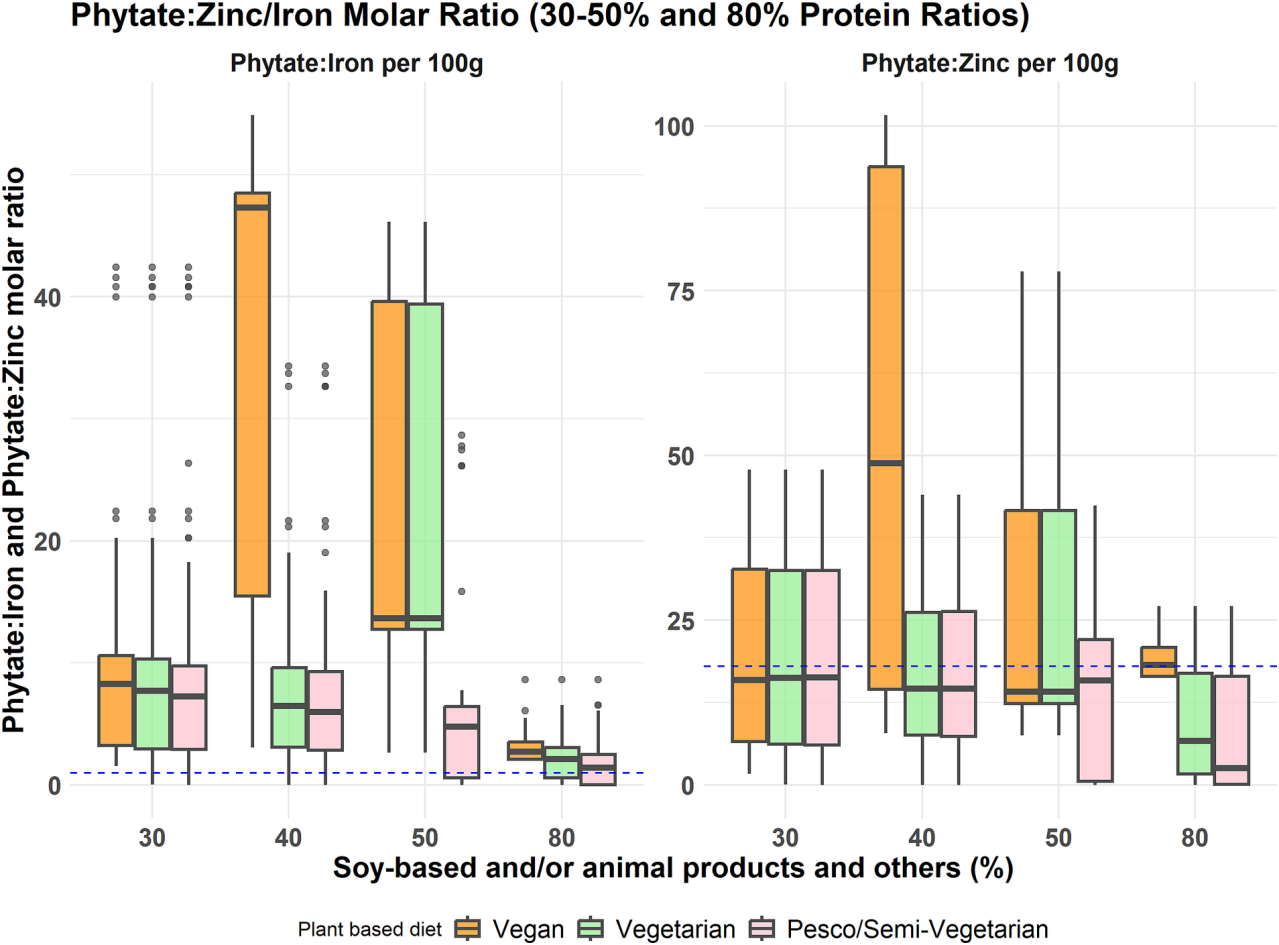
Phytate to iron and phytate to zinc molar ratio distribution across the three plant-based meal models at different protein food contributions. These boxplots display the minimum, interquartile range, median, and maximum phytate to iron molar ratio and phytate to zinc molar ratio levels for the percentage contribution of soy-based and/or animal products to total protein intake across three primarily plant-based meals: vegan, vegetarian, and pesco/semi-vegetarian. The left whisker indicates the minimum contribution, while the upper and lower whiskers represent the lower and upper 25% of intake values, respectively. The box shows the interquartile range, encompassing the middle 50% of intakes (25th to 75th percentile), with the median marked by a line within the box. The right whisker denotes the maximum contribution, and any outliers are displayed as black dots outside the whiskers. The blue dashed line indicates the threshold for phytate-to-iron (<1:1) and phytate-to-zinc ratio (<18:1).

The highest phytate-to-iron and phytate-to-zinc ratios resulted from the vegan meal model, with a 40% contribution from soy-based foods, indicating higher levels of phytate present in comparison to iron and zinc (Figure 4). Notably, all three plant-based meal models had a consistently lower phytate-to-iron and phytate-to-zinc ratio when there was a 30% contribution from soy-based foods alone or the inclusion of animal protein foods. However, none of the plant-based meals met the phytate-to-iron molar ratio threshold (<1:1) for optimal iron absorption. The phytate-to-zinc molar ratio across all plant-based meals was below the threshold for adverse zinc absorption (1, 18), except for the vegan meals with 40 and 80% soy food contribution.

**Figure 5:**
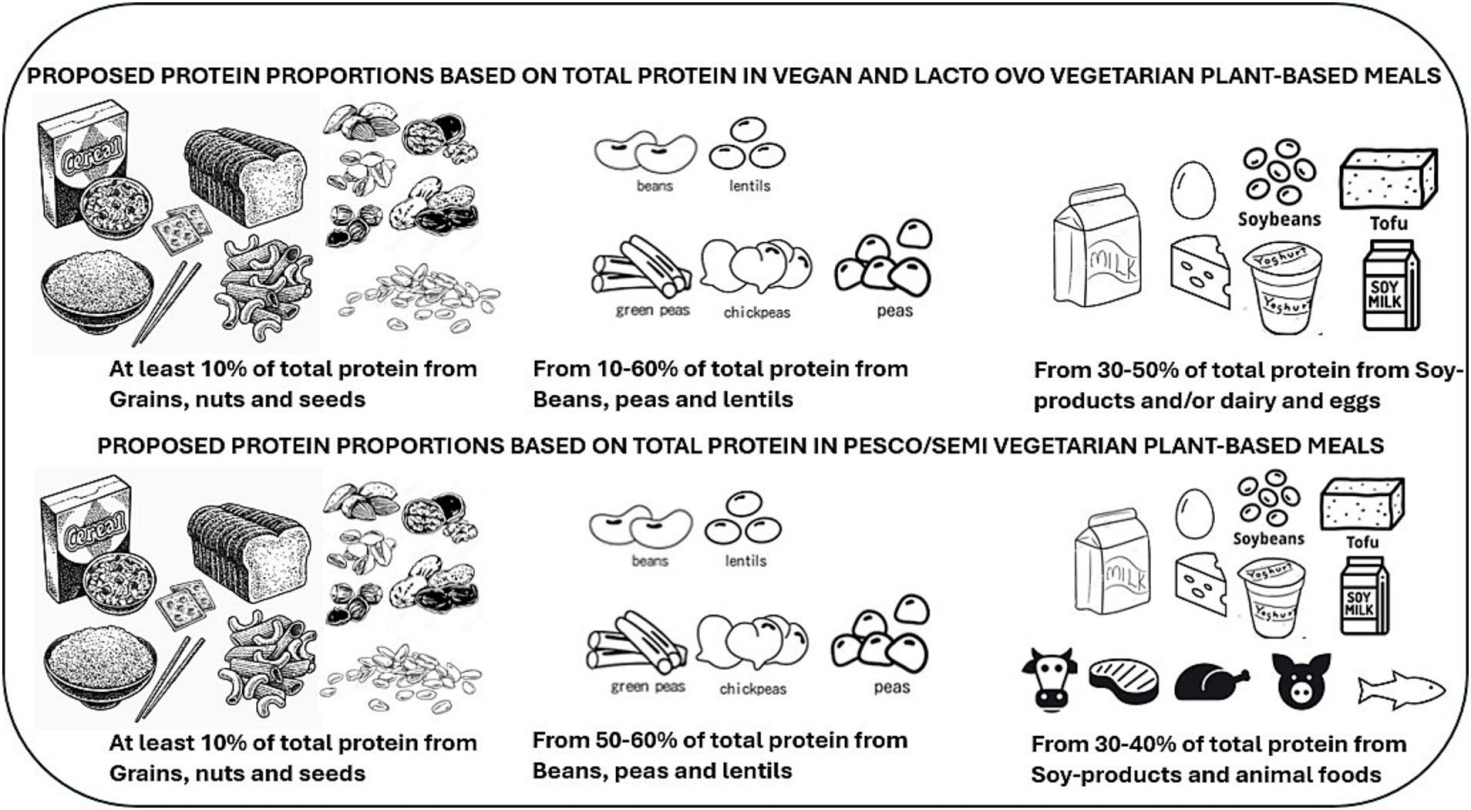
An overview of protein food recommendations that incorporates a diversity of protein food sources needed across various plant-based meals to achieve optimal protein as well as nutrient quality.

Results from the non-optimization model showed that when a meal was modeled to be vegan or vegetarian, the optimal ratio guidance from protein foods could be at least 10% coming from grains, nuts, and seeds, 10 to 60% from beans, peas, and lentils, and 30 to 50% from soy-based foods only and/or dairy and eggs. These protein ratios met all nutrient constraints while providing the highest protein quality for a plant-based meal (Figure 5; Table 5). However, given the low levels of iron and calcium at a 50% contribution from soy and/or animal protein in pesco/semi-vegetarian meals, the optimal protein ratio should consist of at least 10% from grains, nuts, and seeds, 50 to 60% from beans, peas, and lentils, and a minimum of 30 to 40% from soy-based foods, dairy, eggs, fish, poultry, and red meats (Figure 5; Table 5). Notably, with at least 30% contribution from soy-based foods or the inclusion of any animal protein, the protein ratio contributions across all three plant-based meal models were able to meet optimal protein quality and nutrient quality.

**Table 5:** Protein food contributions to total protein meeting all nutrient constraints^a^.

Plant-meal model description	Vegan (plant-foods only)			Vegetarian (dairy and egg included)			Pesco/Semi-vegetarian (dairy, egg, fish, poultry and red-meats included)		
Soy-based/animal-based foods (%)	Solutions (*n*)	Grains, Nuts, Seeds (%)	Beans, Peas, Lentils (%)	Solutions (*n*)	Grains, Nuts, Seeds (%)	Beans, Peas, Lentils (%)	Solutions (*n*)	Grains, Nuts, Seeds (%)	Beans, Peas, Lentils (%)
10	25	10	80	41	10	80	81	10	80
20	85	10	70	111	10	70	168	10	70
**30**	**80**	**10**	**60**	**115**	**10**	**60**	**158**	**10**	**60**
**40**	**55**	**50**	**10**	**78**	**10**	**50**	**100**	**10**	**50**
**50**	**32**	**40**	**10**	**30**	**40**	**10**	41	10	40
60	14	18	22	19	10	30	30	10	30
70	13	16	14	29	10	20	45	10	20
80	19	10	10	41	10	10	59	10	10

^a^Nutrient constraints: Energy (Kcal) ≥ 95–500, Protein (%) ≥ 3.2–30, Dietary fiber (g) ≥ 1.5–5, Iron (mg) ≥ 0.9–2.8, Calcium (mg) ≥ 79–250, Zinc (mg) ≥ 0.7–2.2.

The optimal proportions from the three protein groups for each meal scenario (“vegan,” “vegetarian” and “pesco/semi-vegetarian”) meeting protein quality and nutrient constraints are emphasized in bold.

Table 6 illustrates examples of protein foods with a proportion contribution to total protein intake meeting optimal protein quality and nutrient quality. Each row in Table 6 represents one plant-based meal solution; for example, 50% of the total protein intake should come from whole wheat bread, 10% from carrots, and 40% from tofu. The vegan meal model had more variety coming from grains, nuts and seeds, beans, peas, and lentils than meals including animal protein foods. With the vegetarian meal, no dairy and eggs were observed in the optimal 50% “soy-based foods, dairy products, eggs and others” solution. Similarly, the model included all animal protein foods with no beef in the 40% “soy-based foods, dairy products, eggs, fish, poultry, red-meats and others” solution. It is important to note that the model did not identify beef as an optimal ingredient for semi-vegetarian meals, as its high levels of iron and zinc per 100 grams exceeded the set nutrient constraints for protein and micronutrient quality. Therefore, beef could still be a viable option for inclusion in a semi-vegetarian meal.

**Table 6:** Example of types of protein food contributions to total protein meeting all nutrient constraints.

Vegan					
Grains, nuts, seeds, and others	%	Beans, peas, lentils, and others	%	Soy-based foods and others	%
Whole wheat bread	10	Carrots	60	Tofu	30
Pumpkin seeds		Apples		Tofu	
Oat		Kale		Soybeans	
Brown rice	50	Green peas	10	Tofu	40
Oat		Chickpeas		Tofu	
Barley		Fava beans		Tofu	
White rice	40	Black beans	10	Tofu	50
Millet		Pigeon peas		Tofu	
Oat		Apple		Tofu	

Vegetarian					
Grains, nuts, seeds, and others	%	Beans, peas, lentils, and others	%	Soy-based foods, dairy products, eggs, and others	%
Almonds	10	Potatoes	60	Milk	30
Chia seeds		Apples		Egg	
Almonds		Lupins		Tofu	
Chia seeds	10	Apple	50	Milk	40
Almonds		Kale		Egg	
Seitan		Mushroom		Tofu	
White rice	40	Fava bean	10	Tofu	50
Brown rice		Potatoes		Tofu	
Millet		Pigeon peas		Tofu	

Pesco/Semi-vegetarian					
Grains, nuts, seeds, and others	%	Beans, peas, lentils, and others	%	Soy-based foods, dairy products, eggs, fish, poultry, red meats, and others	%
Almonds	10	Kale	60	Pork	30
Chia seeds		Potatoes		Fish	
Chia seeds		Mushrooms		Eggs	
Oat		Potatoes		Milk	
Almond	10	Chickpea	50	Egg	40
Chia seeds		Carrots		Pork	
Almonds		Kale		Chicken	
Seitan		Potatoes		Milk	

Each row represents one meal combination.

## 4 Discussion

### 4.1 The protein quality and nutritional composition across the protein ratios

Current dietary recommendations from various populations guide the increased variety of plant-protein foods without specifying proportions from each type of plant protein (8–11). Our study provides insights into the diversity of protein foods needed across different plant-based meals to achieve optimal protein as well as nutrient quality. Our findings indicate that a contribution of 30 to 50% from soy-based foods for vegan meals and/or the inclusion of dairy foods and eggs for vegetarian meals presented optimal protein quality (PDCAAS 1) and had the highest levels of calcium and iron (Figures 2, 3). In the case of pesco/semi-vegetarian meals, a contribution of 50% from soy and/or animal protein foods contained the lowest levels of these nutrients, consequently, this proportion was not considered optimal for meals including more animal protein foods. This guidance on protein proportions in plant-based meals could support future dietary guidelines by helping plant-based eaters meet their protein needs, including essential nutrients such as calcium, zinc, and iron (Figure 5).

Overall, at least 30% of either plant-only foods or the inclusion of animal protein foods was required to reach optimal protein and nutrient quality across the different plant-based meals (Figures 2–4). The protein ratio guidance of consuming at least 30% high-quality protein foods such as soy-based foods only and/or animal-based foods is in line with another study’s finding on healthier diets providing adequate nutrients and minimal health risk at a plant protein percentage range from 25 to 70% [53]. Another study in Canada had similar findings and found a meal to be nutritionally balanced when plant protein foods contributed 25–74.9% of the total protein intake [54]. Despite these findings, it is essential to emphasize that the optimized vegan and vegetarian meals could still be low in eicosapentaenoic acid (EPA), docosahexaenoic acid (DHA), iodine, vitamin B12, and vitamin D as unfortified plant protein foods have minimal to none of these nutrients present (30–33). Therefore, including some animal protein in dietary recommendations for vegetarian or pesco/semi-vegetarian meals could aid in increasing the intake of these nutrients found low in plant-based meals, leading to a nutritionally balanced meal. Although several studies have found the vegan diet to have the highest diet quality in comparison to other plant-based diets (34, 35), this was mostly attributed to higher scores from an increased intake of fruits, vegetables, whole grains, plant-protein, fatty acid profile, and lower saturated fat. The diet quality indices used in these studies did not consider the diversity of protein foods consumed or assess whether the intake of any nutrients was low or missing. Therefore, future work should include protein food diversity and consider key nutrient intakes such as iron, calcium, zinc, EPA, DHA, iodine, vitamin B12, and vitamin D when assessing diet quality in plant-based meals.

Other optimization studies for protein quality have been conducted to define optimal plant ingredient combinations that achieve a desirable amino acid profile or high-quality protein meals. For instance, a study based in the Netherlands developed a meal protein scoring system to create high-protein quantity and quality of strict plant-based meals based on indispensable amino acids and digestibility of foods aimed specifically for older adults (36). In contrast, our study findings are applicable for the general population plant-based dieters who are looking to reduce their consumption of animal protein whilst maintaining nutrient quality in a meal, which is a novelty of this study. Another study conducted in France uses a linear programming technique to determine the optimal plant-based ingredient combinations for potential plant-based substitutes. This study results in a combination of plant ingredients meeting three distinct amino acid profiles: first, a ‘balanced amino acid profile’ based on the amino acid requirements for adults; second, ‘animal profiles’ reflecting the compositions of typical animal proteins; and third, a ‘cardioprotective profile’ linked to a reduced risk of cardiovascular disease (37). However, our study takes a different approach by using the PDCAAS as the primary measure of protein quality. Additionally, our study considered key nutrient contributions and examined how phytate may affect the absorption of key minerals such as iron and zinc in plant-based meals.

This study’s vegan and vegetarian meal solutions included a variety of plant protein foods within grains, nuts, seeds, beans, peas, and lentils, unlike the pesco/semi-vegetarian meals, including fish, poultry, and red meats. The increased variety could be due to the need for various plant protein foods to address the limiting amino acids in different plant proteins (38, 39). In contrast, when animal protein foods such as fish, poultry, and red meats were added to the pesco/semi-vegetarian meal model, the optimal ratios differed, with a higher percentage coming from beans, peas, and lentils. Such differences could be due to the constraints on nutrients such as dietary fiber, iron, and calcium in the optimization model. To meet the nutrient constraints included in the optimization model for proposing protein ratios in meals containing animal protein foods such as dairy, eggs, fish, poultry, and red meats, which are high in iron and calcium but lack dietary fiber, the solution was increasing the proportion of beans, peas, and lentils. This solution aims to compensate for the absence of dietary fiber in animal foods while maintaining high protein quality in a meal.

### 4.2 The presence of phytate and selecting the ideal protein ratio

Apart from being important sources of nutrients, plant protein foods such as whole grains, nuts, beans, legumes, and some vegetables contain high amounts of antinutrients, mainly phytates, tannins, lectins, and oxalates (26). Antinutrients like phytates could inhibit protein and mineral absorption. Thus, it is primarily a concern in plant-based meals or diets, which are mainly made up of plant-protein foods (35). The protein foods assessed in this study, such as walnuts, oats, peanuts, and buckwheat, contributed the most phytate from 1,385–4,029 mg per 100 g food (Supplementary Table 1). Our study considered one potential aspect affecting the bioavailability of protein and micronutrients by estimating the phytate levels present in the identified ideal protein ratios. To mitigate excessive amounts of phytates the meal should contain only limited amounts of grains, nuts, and seeds (at least 10% of total protein intake). In the vegan meal model, the molar ratios of phytate to iron and zinc were highest at 50% grains, nuts, and seeds, 10% beans, peas, and lentils, and 40% soy-based foods contribution, indicating a potential decreased bioavailability of iron and zinc at this ratio (Figure 4). Across all plant-based meals, the ratios of phytate to iron and zinc were consistently lowest at 30% soy-based foods only and/or animal protein foods contribution in comparison to all proportions from soy/animal foods, suggesting that this ratio may represent the most effective solution for addressing the presence of antinutrients in plant protein foods. However, it is important to note that across all protein food proportions in all plant-based meals, the phytate-to-iron ratio did not meet the threshold for optimal iron absorption. Therefore, iron absorption enhancers such as vitamin C rich foods, known to counteract the effect of iron absorption inhibitors, should be encouraged when incorporating these proportions into a plant-based meal (29). In our model, enhancers of iron absorption were not considered. However, reasonable consumption of animal protein foods such as meats and fish in a pesco/semi-vegetarian diet could help mitigate the effects of antinutrient factors (40). Additionally, in line with these recommendations from our findings, recent reports indicate that a 30% inclusion of soy in the diet does not significantly impair total iron absorption (41). Further research is needed to evaluate this aspect on mineral absorption, particularly concerning zinc.

### 4.3 The application of an ideal ratio of sources of proteins into dietary guidance

Across all plant-based meals (vegan, vegetarian, and pesco/semi-vegetarian), a consistent finding for achieving optimal protein and nutrient quality was the recommended protein ratio: a minimum of 10% from grains, nuts, and seeds; 60% from beans, peas, and lentils; and 30% from soy-based foods and/or animal protein foods. These ratios also have the most consistent phytate-to-iron and phytate-to-zinc ratios, and if coupled with consuming vitamin C rich foods, this meal proportion could be the most optimal across all plant-based meals. The vegan meal model exhibited the highest diversity among food groups, featuring a broader selection of grains, nuts, seeds, beans, peas, and lentils compared to models that included animal protein foods. Furthermore, a recent review suggests that greater diversity within food groups may enhance nutritional quality and health outcomes (42). With the availability of more data on protein digestibility of foods in the future, this aspect of dietary diversity, both within and between food groups utilizing these ideal protein ratios, will need further investigation to assess its significance for diet quality and health in different study populations.

### 4.4 Strengths and limitations

To our knowledge, this is the first study to use this unique approach to group protein foods by their limiting acids followed by adding nutrient constraints to determine optimal protein and nutrient quality across the different primarily plant-based meals. Furthermore, cooked foods were included in the analysis whenever data was available to account for potential differences between raw and cooked protein foods. The use of secondary data to compile information on amino acid profiles, macronutrients, and micronutrients of the foods included in this study leverages existing USDA datasets that provide complete information on each food item related to protein, amino acids, and nutrients. Another strength to this study was the amount of phytate estimated in the meals to determine if the bioavailability of iron and zinc would be affected across the different primarily plant-based meals. The key findings from this study adds to current knowledge on plant-based dietary guidelines by providing understanding into the types and proportions of protein foods to consume in a plant-based meal.

As the USDA dataset provided comprehensive information on amino acids but lacked data on the protein digestibility of foods, the literature was used to obtain the necessary information for calculating the PDCAAS of each food. The use of different data sources could lead to inconsistencies in the data and the final PDCAAS values. However, due to the absence of a complete data set for protein digestibility of foods, this approach represents the most optimal method for determining the PDCAAS of the foods included in this study. Other limitations of this study include the paucity of *in vivo* data on protein digestibility of protein foods. The PDCAAS is less precise in assessing protein quality than the Digestible Indispensable Amino Acid Score (DIAAS) method. However, as the data on DIAAS for a variety of foods remains limited, the PDCAAS is the most appropriate measurement for protein quality. Future studies could implement a similar model using DIAAS as the objective function instead, leading to an increased accuracy on protein quality measurement. Additionally, this study used the available nutrient guidelines proposed by the International Breakfast Initiative to address the nutrients needed for a plant-based meal. Future studies could adapt to potential developed thresholds for what an ideal protein proportion would be for lunches or dinners, particularly across cultural contexts. This study could not assess vitamin D intakes due to the lack of available data on vitamin D in selected foods, which may have overlooked key differences across the different primarily plant-based meals. Additionally, we included an estimation of phytate levels in foods based on the FAO/INFOODS/IZiNCG Global Food Composition Database for phytate; however, this may not accurately reflect the actual phytate levels in the foods. Finally, enhancers of mineral absorption would also need to be considered in future modeling approaches.

## 5 Conclusion

The main findings from this study provide comprehensive easy-to-follow guidance for selecting a variety of protein foods providing high protein quality while also contributing to nutrient quality in a balanced plant-based meal. These protein food proportions could inform future dietary guidelines by enhancing current recommendations, which primarily emphasize variety in protein sources. The optimal protein ratio from protein foods in vegan meals should be at least 10% grains, nuts, and seeds, 10–60% beans, peas and lentils and 30–50% soy-based foods with the inclusion of dairy and/or eggs in vegetarian meals. As for a pesco/semi-vegetarian meal, the optimal protein ratio should be at least 10% grains, nuts, seeds, 50–60% beans, peas, and lentils, and 30–40% soy-based foods and/or the inclusion of animal protein foods. To reduce animal protein intake in meals, the guidance could be at least 30% of protein foods coming from animal protein foods to be sufficient to meet optimal protein and nutrient quality in a plant-based meal.

## Data Availability

The original contributions presented in the study are included in the article/Supplementary material, further inquiries can be directed to the corresponding author.
