# Supplementary material for: Identifying the optimal ratio from protein foods for protein and nutrient quality in plant-based meals using a non-linear optimization approach
Source: Front Nutr. 2025 Oct 1;12:1624633. doi: 10.3389/fnut.2025.1624633 (PMC12520914; doi:10.3389/fnut.2025.1624633)
Supplement: Supplementary file 1 [file Image_1.pdf]

Identifying the optimal ratio from protein foods for protein and nutrient quality in plant-based meals using a non-linear optimization approach

Maryann Regina Rolands

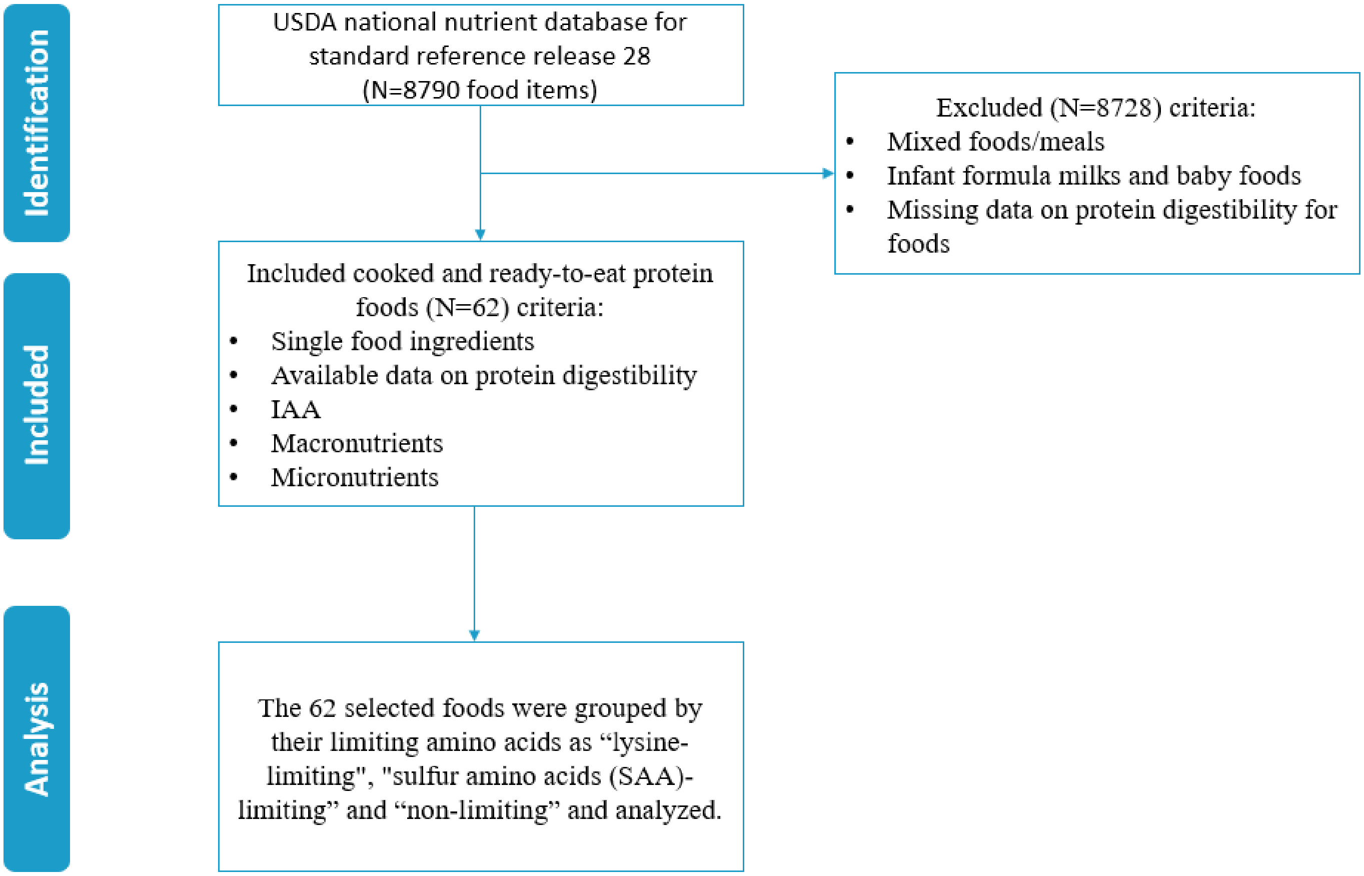

Supplementary Figure 1. Flowchart to demonstrate the selection process for the food data included in the study analysis.
