# Supplementary material for: Identifying the optimal ratio from protein foods for protein and nutrient quality in plant-based meals using a non-linear optimization approach
Source: Front Nutr. 2025 Oct 1;12:1624633. doi: 10.3389/fnut.2025.1624633 (PMC12520914; doi:10.3389/fnut.2025.1624633)
Supplement: Supplementary file 3 [file Table_1.pdf]

Identifying the optimal ratio from protein foods for protein and nutrient quality in plant-based meals using a non-linear optimization approach

Maryann Rolands

Limiting a	Ingredient name	Protein co	Energy pe	Carb	Sugar	Fibre	Fat	Sat fat	Iron	Calcium	Vitamin B	Sodium	Zinc	Sum of aa	Isoleucine	Leucine (r	Lysine (m	Methionin	Phenylala	Threonine	Tryptopha	Valine (m	Histidine	AAS	Protein di	PDCAAS	Phytate (m	Phytate (n	Zinc (mol)	Iron (mol)	Phyt:ZN	Phyt:FE
Non-limiti	Pork, cured, bacon, pre-sliced	33,92	468,00	1,70	0,00	0,00	35,09	11,96	0,95	11,00	1,09	1684,00	3,06	33,66	1,60	1,37	1,74	1,71	2,01	1,79	1,83	1,28	2,58	1,28	98	1,26	0	0	0	0	0	0
Non-limiti	Salami, Italian, pork	21,70	425,00	1,20	1,20	0,00	37,00	13,10	1,52	10,00	2,80	1890,00	4,20	22,40	1,61	1,19	1,75	1,47	1,77	1,81	1,71	1,25	1,71	1,19	96	1,14	0	0	0	0	0	0
Non-limiti	Cheese, gouda	22,87	404,00	2,22	2,22	0,00	27,44	17,61	0,14	710,00	1,10	653,00	3,64	29,38	1,48	1,43	1,88	1,44	2,40	1,27	1,82	1,54	2,20	1,27	88,5	1,12	0	0	0	0	0	0
Non-limiti	Milk, dry, nonfat, calcium redu	35,50	354,00	51,80	0,00	0,00	0,20	0,12	0,32	280,00	3,98	2280,00	4,03	37,27	1,92	1,53	1,58	1,42	2,24	1,72	2,04	1,60	1,61	1,42	92	1,31	0	0	0	0	0	0
Non-limiti	Yeast (Saccharomyces cerevisi	40,44	325,00	41,22	0,00	26,90	7,61	1,00	2,17	30,00	0,07	51,00	7,94	38,34	1,64	1,25	1,78	1,24	1,83	2,08	2,13	1,51	1,48	1,24	82,9	1,02	34,00	0,05	0,12	0,04	0,42	1,33
Non-limiti	Tofu, fried	18,82	270,00	8,86	2,72	3,90	20,20	2,92	4,87	372,00	0,00	16,00	1,99	16,62	1,71	1,29	1,42	1,20	2,07	1,69	2,44	1,30	1,88	1,20	89,64	1,07	301,02	0,46	0,03	0,09	14,98	5,23
Non-limiti	Pork loin, fresh, backribs, bon	24,15	255,00	0,00	0,00	0,00	17,65	6,22	0,96	44,00	0,75	98,00	3,24	23,77	1,60	1,37	1,88	1,71	2,01	1,79	1,83	1,28	2,58	1,28	98	1,26	0	0	0	0	0	0
Non-limiti	Pork, fresh, leg (ham), shank h	25,96	232,00	0,00	0,00	0,00	13,42	4,61	0,86	15,00	0,50	81,00	2,42	25,56	1,60	1,37	1,74	1,71	2,01	1,79	1,83	1,28	2,58	1,28	99	1,27	0	0	0	0	0	0
Non-limiti	Beef, loin, top loin steak, bone	28,57	223,00	0,00	0,00	0,00	11,15	4,53	3,41	14,00	4,25	59,00	4,66	29,21	1,52	1,38	1,95	1,58	1,85	1,84	1,66	1,22	2,29	1,22	98	1,20	0	0	0	0	0	0
Non-limiti	Fish, whitefish, mixed species,	24,47	172,00	0,00	0,00	0,00	7,51	1,16	0,47	33,00	0,96	65,00	1,27	23,61	1,59	1,38	1,98	1,82	1,84	1,82	1,76	1,34	1,91	1,34	91	1,22	0	0	0	0	0	0
Non-limiti	Soybeans, mature cooked, boi	18,21	172,00	8,36	3,00	6,00	8,97	1,30	5,14	102,00	0,00	1,00	1,15	17,61	1,53	1,26	1,31	1,21	2,08	1,64	2,08	1,18	1,59	1,18	94	1,11	0,22	0,00	0,02	0,08	0,02	0,00
Non-limiti	Chicken, broiler or fryers, brea	32,06	157,00	0,00	0,00	0,00	3,24	1,01	0,49	6,00	0,20	47,00	0,96	31,09	1,69	1,40	2,07	1,64	1,92	1,85	1,97	1,33	2,40	1,33	92	1,23	0	0	0	0	0	0
Non-limiti	Egg, whole, cooked, hard-boil	12,60	155,00	1,12	1,12	0,00	10,60	3,27	1,19	50,00	1,11	124,00	1,05	12,57	1,82	1,41	1,50	2,37	2,29	1,92	1,84	1,53	1,48	1,41	89,4	1,26	0	0	0	0	0	0
Non-limiti	Beef, cured, dried	31,10	153,00	2,76	2,70	0,00	1,94	0,95	2,42	8,00	1,59	2790,00	4,93	27,97	1,56	1,34	1,81	1,74	1,79	1,64	1,02	1,27	2,05	1,02	98	1,00	0	0	0	0	0	0
Non-limiti	Cowpeas, catjang, mature see	8,13	117,00	20,30	0,00	3,60	0,71	0,19	3,05	26,00	0,00	19,00	1,87	8,06	1,37	1,27	1,42	1,11	2,23	1,53	1,88	1,20	1,95	1,11	98	1,09	496,10	0,75	0,03	0,05	26,28	13,76
Non-limiti	Buckwheat groats roasted, co	3,38	92,00	19,90	0,90	2,70	0,62	0,13	0,80	7,00	0,00	4,00	0,61	3,06	1,38	1,14	1,17	1,45	1,56	1,69	2,43	1,41	1,62	1,14	88,18	1,00	1397,30	2,12	0,01	0,01	226,91	147,79
Non-limiti	Tofu, silken, extra firm	7,40	55,00	2,00	0,99	0,10	1,90	0,30	1,19	31,00	0,00	63,00	0,60	7,40	1,73	1,42	1,39	1,30	2,45	1,67	2,52	1,35	1,54	1,30	95	1,23	166,38	0,25	0,01	0,10	20,60	2,63
Non-limiti	Soya milk/Soymilk, original an	21,83	54,00	6,28	3,99	0,60	1,75	0,21	0,64	25,00	0,00	51,00	0,12	20,02	1,78	1,38	1,27	1,39	2,10	1,64	4,31	1,30	1,90	1,27	92	1,17	38,19	0,06	0,00	0,01	31,53	5,05
Lysine	Walnuts, english	15,23	654,00	13,70	2,61	6,70	65,20	6,13	2,91	98,00	0,00	2,00	3,09	15,77	1,32	1,22	0,56	1,22	1,73	1,51	1,63	1,19	1,55	0,56	86,22	0,48	1385,00	2,10	0,05	0,05	44,40	40,27
Lysine	Nuts, walnuts, black, dried	24,06	619,00	9,58	1,10	6,80	59,30	3,48	3,12	61,00	0,00	2,00	3,37	24,70	1,30	1,12	0,60	1,64	1,81	1,17	1,95	1,29	1,70	0,60	86,22	0,52	4029,00	6,10	0,05	0,05	118,43	130,79
Lysine	Nuts, almond butter, plain, wi	20,96	614,00	18,80	4,43	10,30	55,50	4,15	3,49	347,00	0,00	7,00	3,29	22,21	1,22	1,09	0,57	0,71	1,92	1,00	1,08	1,05	1,55	0,57	88,90	0,51	566,00	0,86	0,05	0,05	17,04	16,43
Lysine	Nuts, cashew butter, plain, wi	17,56	587,00	27,60	0,00	2,00	49,40	9,76	5,03	43,00	0,00	15,00	5,16	19,34	1,44	1,25	1,01	1,44	1,86	1,40	2,13	1,54	1,48	1,01	87,66	0,89	566,00	0,86	0,08	0,08	10,87	11,40
Lysine	Sunflower seed kernels, dried	20,78	584,00	20,00	2,62	8,60	51,50	4,46	5,25	78,00	0,00	9,00	5,00	25,00	1,52	1,09	0,78	1,64	1,79	1,48	2,11	1,31	1,58	0,78	90,00	0,70	423,00	0,64	0,08	0,09	8,38	6,82
Lysine	Nuts, almonds, whole, raw	21,15	579,00	20,00	0,00	12,50	51,50	3,78	3,71	269,00	0,00	1,00	3,12	22,57	1,11	1,07	0,52	0,72	1,71	1,07	1,42	0,95	1,49	0,52	88,90	0,47	350,00	0,53	0,05	0,07	11,11	7,98
Lysine	Seeds, sesame seed kernels, t	16,96	567,00	26,00	0,48	16,90	48,00	6,72	7,78	131,00	0,00	39,00	10,23	19,22	1,27	1,11	0,59	2,04	2,04	1,46	2,92	1,23	1,62	0,59	75,00	0,44	0,00	0,00	0,00	0,00	0,00	0,00
Lysine	Peanuts, all types, raw	25,80	567,00	16,10	4,72	8,50	49,20	6,28	4,58	92,00	0,00	18,00	3,27	26,06	1,16	1,05	0,74	1,08	2,27	1,36	1,45	1,04	1,56	0,74	90,90	0,67	1994,65	3,02	0,05	0,08	60,43	36,85
Lysine	Pumpkin and squash seed ker	30,23	559,00	10,70	1,40	6,00	49,00	8,66	8,82	46,00	0,00	7,00	7,81	33,45	1,28	1,19	0,77	1,22	2,06	1,19	2,61	1,18	1,46	0,77	90,00	0,69	24,25	0,04	0,12	0,16	0,31	0,23
Lysine	Seeds, watermelon seed kern	28,30	557,00	15,30	0,00	0,00	47,40	9,78	7,28	54,00	0,00	99,00	10,24	31,80	1,40	1,11	0,58	1,74	2,34	1,40	1,86	1,23	1,52	0,58	63,00	0,37	970,00	1,47	0,16	0,13	9,38	11,27
Lysine	Nuts, cashew nuts, raw	18,22	553,00	30,20	5,91	3,30	43,80	7,78	6,68	37,00	0,00	12,00	5,78	20,02	1,31	1,21	0,97	1,64	1,78	1,37	2,17	1,37	1,42	0,97	87,66	0,85	929,00	1,41	0,09	0,12	15,92	11,77
Lysine	Seeds, chia seeds, dried	16,54	486,00	42,10	0,00	34,40	30,70	3,33	7,72	631,00	0,00	16,00	4,58	19,49	1,37	1,15	1,04	2,22	1,98	1,46	3,39	1,22	1,70	1,04	29,00	0,30	0,00	0,00	0,07	0,12	0,00	0,00
Lysine	Oat	16,89	389,00	66,30	0,00	10,60	6,90	1,22	4,72	54,00	0,00	2,00	3,97	16,78	1,38	1,25	0,87	1,87	2,13	1,37	2,11	1,40	1,51	0,87	91,00	0,79	991,00	1,50	0,06	0,08	24,73	17,77
Lysine	Fried seitan coated with whea	20,93	359,00	61,20	0,50	8,90	7,23	1,03	4,24	63,00	0,00	282,00	2,93	19,16	1,32	1,21	0,32	1,79	1,93	0,96	4,51	1,02	1,53	0,32	97,50	0,31	266,00	0,40	0,04	0,08	8,99	5,31
Lysine	Cereals ready-to-eat, KELLOG(	7,50	357,00	84,10	9,50	3,30	0,40	0,12	28,90	5,00	0,00	729,00	1,00	6,91	1,21	2,48	0,24	1,71	1,60	1,31	0,99	1,11	1,54	0,24	67,00	0,16	46,20	0,07	0,02	0,52	4,58	0,14
Lysine	Bread, whole-wheat, prepare	8,40	278,00	51,40	3,84	6,00	5,40	0,80	3,10	33,00	0,00	346,00	1,50	8,39	1,25	1,12	0,61	1,67	1,90	1,18	2,20	1,12	1,45	0,61	91,00	0,55	330,85	0,50	0,02	0,06	21,85	9,03
Lysine	Bread, pita, white, unenriched	9,10	275,00	55,70	0,00	2,20	1,20	0,17	1,40	86,00	0,00	536,00	0,84	9,05	1,28	1,15	0,50	1,71	1,89	1,14	1,76	1,09	0,63	0,50	94,73	0,48	0,00	0,00	0,00	0,00	0,00	0,00
Lysine	Oat bran, raw	17,30	246,00	66,20	1,45	15,40	7,03	1,33	5,41	58,00	0,00	4,00	3,11	17,79	1,25	1,27	0,89	2,23	2,16	1,13	2,85	1,35	1,44	0,89	74,00	0,66	2769,00	4,20	0,05	0,10	88,20	43,31
Lysine	Tortillas, ready-to-bake or -fry	5,70	218,00	44,60	0,88	6,30	2,85	0,45	1,23	81,00	0,00	45,00	1,31	5,79	1,20	2,01	0,59	1,70	2,19	1,51	1,10	1,27	1,91	0,59	84,00	0,49	1073,00	1,63	0,02	0,02	81,14	73,81
Lysine	Nuts, coconut milk, canned (li	2,02	197,00	2,81	0,00	0,00	21,30	18,90	3,30	18,00	0,00	13,00	0,56	2,20	1,20	1,12	0,84	1,54	1,82	1,34	1,65	1,39	1,31	0,84	54,00	0,45	0,00	0,00	0,00	0,00	0,00	0,00
Lysine	Pasta, whole-wheat, cooked	5,99	149,00	30,10	0,75	3,90	1,71	0,24	1,72	13,00	0,00	4,00	1,34	5,98	1,29	1,12	0,46	1,62	1,85	1,08	1,95	1,08	1,46	0,46	92,00	0,43	0,07	0,00	0,02	0,03	0,01	0,00
Lysine	Rice, white, long-grain, regula	2,69	130,00	28,20	0,05	0,40	0,28	0,08	0,20	10,00	0,00	1,00	0,49	2,69	1,44	1,35	0,75	1,91	2,12	1,43	1,75	1,53	1,46	0,75	72,80	0,55	60,00	0,09	0,01	0,00	12,13	25,38
Lysine	Barley pearl cooked	2,26	123,00	28,20	0,28	3,80	0,44	0,09	1,33	11,00	0,00	3,00	0,82	2,26	1,22	1,12	0,77	1,79	2,07	1,36	2,55	1,23	1,41	0,77	78,00	0,60	30,70	0,05	0,01	0,02	3,71	1,95
Lysine	Rice, brown, long-grain, cooke	2,74	123,00	25,60	0,24	1,60	0,97	0,26	0,56	3,00	0,00	4,00	0,71	2,58	1,41	1,36	0,80	1,50	2,17	1,47	1,94	1,46	1,60	0,80	84,44	0,67	612,16	0,93	0,01	0,01	85,41	92,49
Lysine	Millet, cooked	3,51	119,00	23,70	0,13	1,30	1,00	0,17	0,63	3,00	0,00	2,00	0,91	3,51	1,41	2,08	0,40	1,70	2,04	1,29	1,64	1,31	1,34	0,40	80,17	0,32	0,43	0,00	0,01	0,01	0,05	0,06
Lysine	Oat bran, cooked	3,21	40,00	11,40	0,00	2,60	0,86	0,16	0,88	10,00	0,00	1,00	0,53	3,04	1,25	1,27	0,89	2,23	2,16	1,13	2,84	1,36	1,44	0,89	87,42	0,78	940,00	1,42	0,01	0,02	175,69	90,38
Met+Cys	Potatoes, french fried, cottage	3,44	218,00	34,00	0,00	3,20	8,20	3,89	1,49	10,00	0,00	45,00	0,41	3,24	1,52	1,05	1,18	0,81	1,75	1,93	2,15	1,35	1,12	0,81	51,00	0,41	106,00	0,16	0,01	0,03	25,61	6,02
Valine	Chickpeas (garbanzo beans, b	8,86	164,00	27,40	4,80	7,60	2,59	0,27	2,89	49,00	0,00	7,00	1,53	8,55	1,48	1,21	1,44	1,19	1,98	1,54	1,51	1,09	1,78	1,09	74,60	0,81	76,93	0,12	0,02	0,05	4,98	2,25
Met+Cys	Beans, pinto, mature seeds, co	9,01	143,00	26,20	0,34	9,00	0,65	0,14	2,09	46,00	0,00	1,00	0,98	8,89	1,60	1,41	1,48	0,98	2,04	1,49	1,84	1,46	1,74	0,98	76,23	0,75	349,67	0,53	0,01	0,04	35,35	14,16
Met+Cys	Beans, navy, mature seeds, co	8,23	140,00	26,00	0,37	10,50	0,62	0,10	2,36	69,00	0,00	0,00	1,03	8,36	1,54	1,37	1,30	0,97	1,95	1,38	1,81	1,51	1,54	0,97	79,96	0,78	0,18	0,00	0,02	0,04	0,02	0,01
Met+Cys	Beans, black, mature seeds, co	8,86	132,00	23,70	0,32	8,70	0,54	0,14	2,10	27,00	0,00	1,00	1,12	8,40	1,55	1,38	1,51	1,19	2,12	1,78	1,89	1,38	1,84	1,19	69,99	0,83	0,18	0,00	0,02	0,04	0,02	0,01
Met+Cys	Beans, adzuki, mature seeds,	7,52	128,00	24,80	0,00	7,30	0,10	0,04	2,00	28,00	0,00	8,00	1,77	7,16	1,40	1,45	1,65	0,91	2,12	1,43	1,52	1,35	1,73	0,91	75,90	0,69	338,00	0,51	0,03	0,04	18,92	14,30
Met+Cys	Beans, kidney, all types, matu	8,67	127,00	22,80	0,32	6,40	0,50	0,07	2,22	35,00	0,00	1,00	1,00	8,22	1,55	1,38	1,51	1,18	2,11	1,78	1,90	1,38	1,84	1,18	74,00	0,88	0,15	0,00	0,02	0,03	0,02	0,01
Met+Cys	Pigeon peas , mature seeds, c	6,76	121,00	23,25	0,00	6,70	0,38	0,08	1,11	43,00	0,00	5,00	0,90	6,75	1,21	1,17	1,46	0,99	2,70	1,42	1,48	1,08	2,23	0,99	71,60	0,71	0,06	0,00	0,02	0,02	0,01	0,00
Met+Cys	Lupins, mature seeds, cooked	15,57	119,00	9,88	0,00	2,80	2,92	0,35	1,20	51,00	0,00	4,00	1,38	15,74	1,47	2,50	1,10	0,83	1,86	1,46	1,20	1,03	1,76	0,83	90,00	0,75	926,45	1,40	0,02	0,02	66,50	65,33
Met+Cys	Peas,green, split, mature seed	8,34	118,00	21,10	2,90	8,30	0,39	0,05	1,29	14,00	0,00	2,00	1,00	7,97	1,44	1,23	1,57	1,16	1,92	1,49	1,77	1,24	1,59	1,16	85,15	0,98	0,10	0,00	0,02	0,02	0,01	0,01
Met+Cys	Lentils, mature seeds, cooked	9,02	116,00	20,10	1,80	7,90	0,38	0,05	3,33	19,00	0,00	2,00	1,27	8,29	1,57	1,29	1,58	1,02	2,02	1,56	1,48	1,35	1,91	1,02	90,60	0,93	316,40	0,48	0,02	0,06	24,68	8,04
Met+Cys	Broad beans (fava beans), ma	7,60	110,00	19,60	1,82	5,40	0,40	0,07	1,50	36,00	0,00	5,00	1,01	7,10	1,44	1,32	1,43	0,97	1,93	1,52	1,54	1,19	1,70	0,97	77,90	0,76	0,16	0,00	0,02	0,03	0,02	0,01
Met+Cys	Mung beans, mature seeds, co	7,02	105,00	19,20	2,00	7,60	0,38	0,12	1,40	27,00	0,00	2,00	0,84	6,81	1,45	1,31	1,50	0,93	2,28	1,35	1,69	1,34	1,88	0,93	63,20	0,59	657,23	1,00	0,01	0,03	77,51	39,72
Leucine	Potatoes, boiled, cooked with	1,71	86,00	20,00	0,89	1,80	0,10	0,03	0,31	8,00	0,00	5,00	0,27	1,72	1,36	0,98	1,26	1,24	1,99	1,45	2,39	1,40	1,38	0,98	95,00	0,94	47,61	0,07	0,00	0,01	17,47	13,00
Met+Cys	Apples, raw, golden delicious,	0,28	57,00	13,60	10,00	2,40	0,15	0,00	0,13	6,00	0,00	2,00	0,04	0,21	0,94	1,08	1,27	0,41	0,92	1,13	0,71	1,53	1,47	0,41	52,00	0,21	0,57	0,00	0,00	0,00	1,41	0,37
Leucine	Carrots, cooked, boiled, drain	0,76	35,00	8,22	3,45	3,00	0,18	0,03	0,34	30,00	0,00	58,00	0,20	1,41	1,07	0,67	0,69	0,91	1,00	1,16	1,36	1,03	1,00	0,67	80,00	0,54	0,00	0,00	0,00	0,00	0,00	0,00
Met+Cys	Kale, cooked, boiled, drained,	1,90	28,00	5,30	1,21	2,00	1,21	0,18	0,90	72,00	0,00	23,00	0,24	1,69	2,24	1,29	1,40	1,10	2,36	2,01	2,06	1,54	1,48	1,10	77,00	0,85	127,00	0,19	0,00	0,02	52,42	11,94
Met+Cys	Mushrooms, portabella, raw	2,11	22,00	3,87	2,50	1,30	0,35	0,01	0,31	3,00	0,00	9,00	0,53	1,79	1,53	1,19	1,42	0,95	1,23	2,26	2,97	1,06	2,03	0,95	81,57	0,77	5,37	0,01	0,01	0,01	1,00	1,47
