# Supplementary material for: Identifying the optimal ratio from protein foods for protein and nutrient quality in plant-based meals using a non-linear optimization approach
Source: Front Nutr. 2025 Oct 1;12:1624633. doi: 10.3389/fnut.2025.1624633 (PMC12520914; doi:10.3389/fnut.2025.1624633)
Supplement: Supplementary file 4 [file Table_2.docx]

Supplemental Table 2. Calculations for PDCAAS for each combination of three protein food sources

| **Parameter** | **Formula** |
| --- | --- |
| Total protein (g/100g) | [(% ingredient1 in combination **×** ingredient1 protein %) + (% ingredient2 in combination **×** ingredient2 protein %) + (% ingredient3 in combination **×** ingredient3 protein %)] |
| Reference IAA  (FAO 2013 for children over 3 years & adults) | Isoleucine (30 mg/g)  Leucine (61 mg/g)  Lysine (48 mg/g)  Methionine + cysteine (23 mg/g)  Phenylalanine + tyrosine (41 mg/g)  Threonine (25 mg/g)  Tryptophan (6.6 mg/g)  Valine (40 mg/g)  Histidine (16 mg/g) |
| Sum of each IAA (mg/g) | [(% ingredient1 in combination **×** IAA (mg/g)/total protein (g/100g) **×** 1000/ reference IAA (mg/g)) + (%ingredient2 in combination **×** IAA (mg/g)/total protein (g/100g) **×** 1000/ reference IAA (mg/g)) + (% ingredient3 in combination **×** IAA (mg/g)/total protein (g/100g) **×** 1000/ reference IAA (mg/g))] |
| Total protein digestibility (%) | [(% ingredient1 protein in combination/total protein (g/100) **×** ingredient1 protein digestibility %) + (% ingredient2 protein in combination/total protein (g/100) **×** ingredient2 protein digestibility %) + (% ingredient3 protein in combination/total protein (g/100) **×** ingredient3 protein digestibility %)] |
| Amino acid score (mg/g) | IAA with the lowest amount |
| PDCAAS | Amino acid score (mg/g) X Total protein digestibility (%) /100 |
